# A “reverse pharmacology” approach for developing an anti-malarial phytomedicine

**DOI:** 10.1186/1475-2875-10-S1-S8

**Published:** 2011-03-15

**Authors:** Merlin L Willcox, Bertrand Graz, Jacques Falquet, Chiaka Diakite, Sergio Giani, Drissa Diallo

**Affiliations:** 1 Department of Primary Health Care, University of Oxford, UK; 2 Research Initiative for Traditional Antimalarial Methods, Oxford, UK; 3 Institut de Médecine Sociale et Préventive, Geneva University, Switzerland; 4 Département de Médecine Traditionnelle, Bamako, Mali; 5 Aidemet NGO, Bamako, Mali

## Abstract

A “reverse pharmacology” approach to developing an anti-malarial phytomedicine was designed and implemented in Mali, resulting in a new standardized herbal anti-malarial after six years of research. The first step was to select a remedy for development, through a retrospective treatment-outcome study. The second step was a dose-escalating clinical trial that showed a dose-response phenomenon and helped select the safest and most efficacious dose. The third step was a randomized controlled trial to compare the phytomedicine to the standard first-line treatment. The last step was to identify active compounds which can be used as markers for standardization and quality control. This example of “reverse pharmacology” shows that a standardized phytomedicine can be developed faster and more cheaply than conventional drugs. Even if both approaches are not fully comparable, their efficiency in terms of public health and their complementarity should be thoroughly considered.

## Background

Malaria elimination efforts will lead to the much wider use of the few currently effective anti-malarial drugs, such as artesunate / amodiaquine, artesunate / sulphadoxine-pyrimethamine (SP), and artemether / lumefantrine. There is already discussion about intermittent presumptive treatment of infants, children, pregnant women, and even mass drug administration in some settings [1]. Resistance already exists to amodiaquine and SP, and will probably increase as a result of the increased drug pressure. The first signs of resistance to artemisinin derivatives are appearing in Cambodia [2].

In this context it is important to maximize the lifespan of existing anti-malarials, and to consider all options for the development of new anti-malarials. Traditional medicinal plants have provided the source of the two major families of anti-malarial drugs still in use today, artemisinin and quinine, so many researchers are screening plants for novel chemical entities to develop as “lead compounds” for new anti-malarial drugs [3]. However conventional drug development is slow and expensive, taking up to 15 years and up to $800m to develop a new drug [4,5]. Furthermore the finished products are often unavailable and unaffordable to the poorest patients in remote areas, unless they are part of a heavily subsidized scheme.

In contrast the parallel development of standardized phytomedicines can be done faster, more cheaply, and more sustainably for remote areas. They could then be proposed and tested as a complement to existing strategies, for example as first aid in remote areas in case there is some delay until ACT treatment can be started. Their use might also delay the development of resistance to current standard drugs. The concept of “reverse pharmacology” was coined in India to develop pharmaceuticals from Ayurvedic medicines, and was also championed by the Chinese in the 1950s [6], but still involved a classical pathway of isolating compounds for further development [7]. The saving in time and cost comes from the fact that substantial experience of human use increases the chances that a remedy will be effective and safe, and that precautions will be known. However, as with classic drug discovery, there is no guarantee of successfully developing new treatments.

In order to develop a standardized phytomedicine, a “reverse pharmacology” approach was tested, where clinical evaluation was prioritized from the start. Isolation of compounds was done only at the end of the pathway, mainly for the purposes of quality control, agronomic selection and standardization, if justified by the clinical results. This experience led to the development of a new anti-malarial phytomedicine from a traditional herbal remedy, namely *Argemone mexicana* decoction, which is in the process of being approved in Mali. The regulatory requirements for herbal medicines are completely different to those for new drugs. It should be emphasized that the primary objective of the project described here was not to develop new drugs, but to improve the utilization of herbal medicines, which are already in use. All the clinical studies described below were reviewed and approved by the Ethics Committee of the Institut National de Recherche en Santé Publique (INRSP) in Mali. This process took six years and cost about 400,000 Euros.

The research project is described here as it actually happened. Some aspects are reviewed in the discussion section, as with the benefit of hindsight some procedures might be improved. The hope is that this paper may help others who are interested in conducting a similar process (developing phytomedicines, perhaps for other indications) through a clear report of what was planned, what was opportunistically added – and what was obtained.

## Stage 1: Selection of a herbal remedy

The classical way of identifying medicinal plants for further research is through ethnobotanical studies. Yet conventional ethnobotanical studies rarely involve clinicians. They could and should provide much more clinical information if the ultimate goal is to know which one, among numerous treatments for a given ailment, has the best effects [8]. Although identification of the plants is usually of a good standard, definition of the diseases which they treat is not. There is rarely sufficient questioning about the observed patient status and progress, perceived efficacy and limitations of the remedies, and whether these are indeed the “treatment of choice”. Many plants are “supposed” to be good for one disease or another, but are not actually the preferred treatment used in everyday life. In order to circumvent these problems, Graz *et al* developed a new method called a “Retrospective Treatment Outcome Study” (RTO) [9]. This simply adds two essential elements to the ethnobotanical method: clinical information and statistical analysis. Clinical information is collected retrospectively on the presentation and progress of a defined disease episode. Treatments and subsequent clinical outcomes are analysed to elicit statistically significant correlations between them. Such an approach requires a large sample if the number of different treatments is high. This method makes it possible to identify the remedy which has the highest statistical correlation with reported clinical recovery. The hypothesis is that this correlation is a marker of effectiveness, which can then be further tested in a prospective clinical study of the selected remedy. It was also hypothesized that if a treatment is often associated with failure, this is a marker of ineffectiveness.

In the RTO, the first step was to understand local concepts and terms for diseases. The aim was to maximize the chances that the respondents were giving information about the disease of interest to researchers. For uncomplicated malaria, the definition was “fever with no other obvious cause during the rainy season” and for severe malaria, it was “fever with convulsions or loss of consciousness during the rainy season”[10]. In Mali, these correspond to the local Bambara terms “soumaya” and “kono” respectively. Of course these are not very precise, but they are the same definitions as those used for presumptive treatment [11], and the best that can be done retrospectively, when blood tests are impossible.

The second step was to choose a representative random sample of households in the study area (by cluster sampling), and to ask in each whether anyone has had the disease of interest in the recent past. The timing was at the end of the malaria season (for example in Mali the rains begin in July and the perfect time for such a study would be in November - December). For uncomplicated malaria, a recall time of two weeks was used (as this is a common event, and there is a risk that information will be inaccurate if the recall period is too long) [10]. For severe malaria (which is rarer and more dramatic, so more likely to be remembered) a recall period of six months was used. The sample size was determined on the basis of the estimated prevalence of malaria in the area, and the estimated number of different treatments (from previous information).

The third step, if a respondent had had the condition of interest, was to ask in detail what treatments they had taken, in what order, at what stage they had recovered from their illness and if the cure was complete or with sequelae. In this way it was possible to understand what treatments patients were actually using in real life and with what results.

In Mali, use of this method resulted in a database of treatments taken for malaria cases in 952 households. The analysis was an iterative process performed with the help of a statistician, starting with a test of correlation between reported clinical outcome and the plant used. Since in some cases recipes contained more than one plant, a second step was to adjust for this in the analysis, in an attempt to determine whether individual components were associated with clinical outcomes. From the 66 plants used for the treatment of malaria in the two districts studied in Mali, alone or in various combinations, the one associated with the best outcomes was a decoction of *Argemone mexicana* (Table 1). The clinical outcomes were not better when it was used in combination [12]. This remedy was selected for further study.

**Table 1:** Sample results from the RTO study for the three most promising plants (the full table included 66 plants in total).

Plant	Preparation	No of cases reporting use	No of cases reporting clinical recovery	No of treatment failures	Correlation with clinical recovery	(95% CI)	P (Fisher) *
*Argemone mexicana* (Papaveraceae)	Aerial parts decoction	30	30	0	100%	(88 - 100)	NA (best results)
*Carica papaya*	Leaves decoction	33	28	5	85%	(68-95)	0.05
*Anogeissus leiocarpus*	Leaves	33	27	6	82%	(64-93)	0.03

*(Number with and without clinical recovery compared to the plant with best results

At this stage, there was the opportunity to test some of the plants for their anti-malarial activity *in vitro* (Table 2). *Argemone mexicana* had the best activity *in vitro*, both for the extracts in polar solvents and the aqueous decoction [12] The IC50 of the methanol extract was 1.0 μg/ml, which is of the same order as the ethanolic extract of *Artemisia annua*[*13*] .

**Table 2:** *In vitro* anti-malarial activity of plant extracts identified in a retrospective treatment-outcome study, for plants with aqueous extracts having IC50 <10 μg/ml [12]

Plant	Plant part	Extract	IC_50_ (μg/ml)
***Argemone mexicana***	Aerial parts	Methanol	1.00
***Argemone mexicana***	Aerial parts	Dichloromethane	1.22
***Argemone mexicana***	Aerial parts	Aqueous decoction	5.89
***Argemone mexicana***	Aerial parts	Aqueous maceration	6.22
***Opilia celtidifolia***	Bark	Aqueous maceration	7.64
***Spondias mombin***	Leaves	Aqueous maceration	7.66
***Securinega virosa***	Leaves	Aqueous decoction	7.81
***Spondias mombin***	Leaves	Aqueous decoction	7.89
***Cassia sieberiana***	Roots	Aqueous maceration	7.93
***Canthium acutiflorum***	Leaves	Aqueous maceration	8.09
***Securinega virosa***	Roots	Aqueous decoction	8.69
***Opilia celtidifolia***	Bark	Maceration in warm water then decoction	9.07
***Feretia apodanthera***	Bark	Aqueous decoction	9.54
***Securinega virosa***	Roots	Aqueous maceration	9.68
***Canthium acutiflorum***	Bark	Aqueous decoction	9.73

Before proceeding to clinical studies, it is important to establish that the remedy is safe. WHO guidelines [14] state that: “*If the product has been traditionally used without demonstrated harm*, *no specific restrictive regulatory action should be undertaken unless new evidence demands a revised risk-benefit assessment.*” WHO maintains the position that there is no requirement for pre-clinical toxicity testing; rather that evidence of traditional use or recent clinical experience is sufficient [15]. Indeed often the same plants are traditionally used both as a food and as a medicine [16], and no toxicological tests are required for foods, which are usually consumed in greater quantities than medicines. Pre-clinical toxicity testing is only required for new medicinal herbal products which contain herbs with no traditional history of use. Therefore, if preliminary field studies (such as the RTO study) have shown that the preparation is of common and ancient use, with no known important side effects, toxicological studies are unnecessary [17].

The literature was searched extensively [18] to see whether the safety of the remedy had already been established in previous studies. The aim was to find studies of the same plant part, using a similar extraction method, to answer the following questions:

1. Are there any reports of human toxicity associated with ingestion of the plant? If so, which part of the plant, in what preparation, at what dose, and what were the consequences?

2. Have any laboratory studies of toxicity been carried out on the relevant preparation of the plant? If so, what did the results show?

3. What pharmacologically active compounds does this plant species contain? In which parts of the plant are they found? What are their principle pharmacological effects, and at what doses?

Search terms included the plant species and major chemical compounds known to exist in the plant (principally berberine for *Argemone mexicana*). None of the existing databases or books can cover all published information on a given topic, and therefore as many sources of information as possible were consulted: firstly freely available online databases [19,20]; then reference books such as pharmacopoeiae and similar monographs [21], and texts on plant toxicology and herbal medicine safety [22-24]; and finally, other databases: EMBASE, CAB Global Health, and the Allied and Complementary Medicine database.

In the case of *Argemone mexicana*, the literature search revealed no toxicology studies but there were reports of “epidemic dropsy” in India attributed to the ingestion of the seed oil containing sanguinarine, as a contaminant in culinary oils [25]. This was of some concern, therefore the traditional healer was asked to remove seed capsules from the preparation used for clinical studies in Mali. However, there were no references to toxicity from an aqueous decoction of the leaves and stems (which was the traditional preparation in question), and this remedy was reported in the ethnobotanical literature as being used in Benin, Mali, India and Colombia [26-29].

## Stage 2: Dose-escalating clinical study

As patients were using the remedy in any case, and the literature search did not reveal any concerns, an observational cinical study was organized with a small number of patients. It is a prerequisite to conduct such a study in an area where patients are already taking the remedy, so that one is not proposing a new treatment (for example for a comparative prospective study – see below) without some clinical evidence of effect size and safety.

The traditional preparation was given to patients with uncomplicated malaria, who met the following criteria:

1. Inclusion criteria:

a. Symptoms of malaria (fever) within the last 24 hours

b. Parasitaemia >2000/mcl and <200 000 / mcl on microscopy

c. Informed consent of patient or parent

2. Exclusion criteria:

a. Signs of severe malaria

b. age <3 months

c. pregnancy

d. other concomitant febrile illness

e. administration of a full course of anti-malarial (modern or traditional) within the previous week

f. inability to return for follow-up.

**Figure 1:**
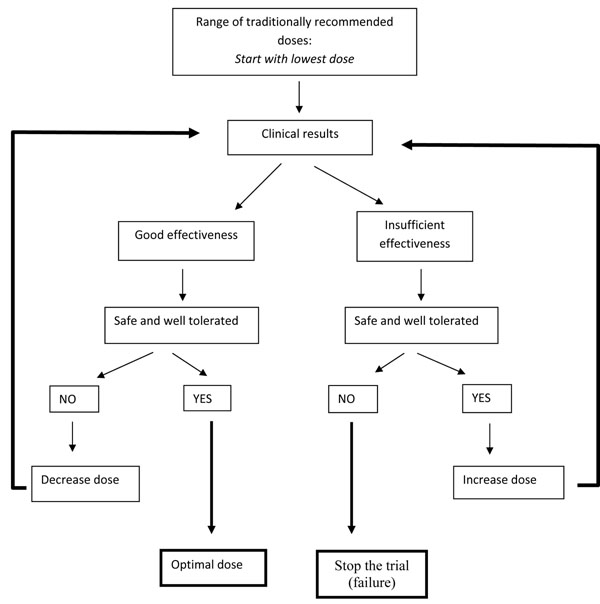
Dose optimization

Patients were followed up closely on days 1, 2, 3, 7, 14 and 28, and were advised to return immediately at any other time if their condition deteriorated. Monitoring included parameters of efficacy (temperature, symptoms, parasite counts) and safety (new symptoms/adverse events, ECGs, and blood tests to monitor bone marrow function, renal function and liver function). The design of the whole study became a sequential follow-up of patients using, in the first group, a dose lower than the one traditionally used (but, at the time, proposed as the correct one by the traditional healer), then the bottom and top of the usual dose range (see Figure 1). In this way patients always received the best dose according to the current state of knowledge. If the incidence of important adverse effects reached an unacceptable level, the trial could of course be stopped. Compliance was monitored by direct observation of some doses of the treatment (the first dose of each of the first three days when the patient attended for follow-up) and by asking patients whether they had taken the recommended dosage during the rest of the day. Thus, it was also possible to assess whether the optimal dosage was realistic and feasible in the field.

**Figure 2:**
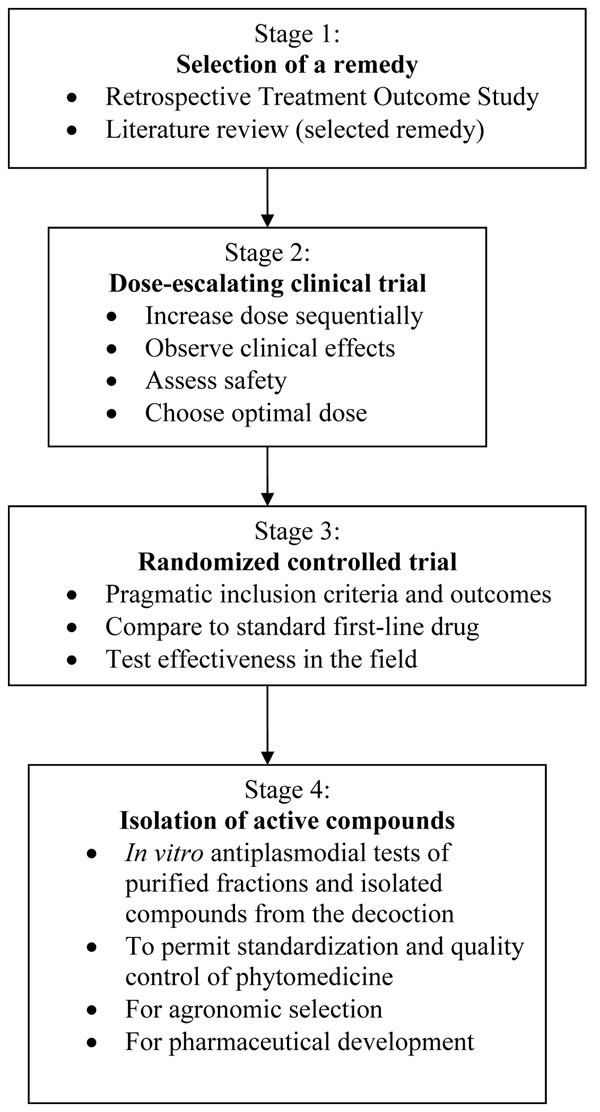
Summary of the methodology used to develop an anti-malarial phytomedicine by “reverse pharmacology”

The outcome measures chosen were appropriate to the context in which use of the phytomedicine was envisaged, which was a high-transmission area (Tables 3 and 4). In low transmission areas, the outcome recommended by WHO is “Adequate Clinical and Parasitological Response” (ACPR) which includes a requirement that the parasite count by day 3 is reduced to <25% of baseline, and that total parasite clearance is achieved by day 7 and maintained through to day 28 [30]. Although WHO now also recommends total parasite clearance in all situations, this may not be necessary in high transmission areas where the population develops partial immunity in early life, and is rapidly re-infected even if parasite clearance is achieved. In such high transmission areas, the most useful outcome measures are clinical rather than parasitological. One such is the rate of “adequate clinical response” (ACR, see table 3), which is a modification of ACPR. The criterion that parasitaemia on day 3 should have decreased to <25% of that on day 0 was designed for fast-acting drugs, such as chloroquine and artemisinin derivatives. It is not an essential criterion for testing slower-acting drugs, such as quinine and herbal remedies, and therefore can be omitted for such trials [31]. If patients clinically worsened or did not recover (i.e. “treatment failures”) they were given an alternative treatment (the nationally recommended anti-malarial).

**Table 3:** Modified classification of treatment outcomes for trials on herbal antimalarials in high transmission areas [31,43]

***Adequate Clinical Response*** **(ACR)** On day 14, without previously meeting any of the criteria of *Early Treatment Failure* or *Late Treatment Failure:* • Either absence of parasitaemia irrespective of axillary temperature • Or axillary temperature <37.5°C and no history of fever in the last 24 hours irrespective of the presence of parasitaemia
***Early Treatment Failure*** **(ETF)** • Development of danger signs or severe malaria on Day 1, Day 2 or Day 3, in the presence of parasitaemia; • Parasitaemia on Day 2 higher than Day 0 count irrespective of axillary temperature; • Parasitaemia on Day 3 with axillary temperature ≥ 37.5 °C; *afebrile patients with parasitaemia on day 3* ≥*25% of count on day 0 will NOT be counted as early treatment failures*, *but will be observed closely*
***Late Treatment Failure*** **(LTF)** • Development of danger signs or severe malaria after Day 3 in the presence of parasitaemia, without previously meeting any of the criteria of *Early Treatment Failure* • Presence of parasitaemia and axillary temperature ≥ 37.5 °C (or history of fever) on any day from Day 4 to Day 14, without previously meeting any of the criteria of *Early Treatment Failure*

The idea of dose escalation was developed by chance. The original intention was to observe a cohort of patients being treated by a traditional healer at a dose decided by the healer. However, it soon became clear that the dose initially chosen by the healer (“A” = one glass a day for 3 days) was insufficient, with an ACR in only 35% of patients. When questioned why he had chosen this dose, the healer replied that he thought it was “more scientific” (perhaps because it is similar to the dosage of chloroquine, which is given once daily for three days). He then revealed that in fact he normally told patients how to prepare the remedy, and advised them to drink as much of it as possible. Therefore two other standard doses were agreed: one glass twice a day for 7 days (B), and one glass 4 times a day for the first 4 days, followed by one glass twice a day up to 7 days (C). Increasing the dose from A to B improved the efficacy (the proportion of patients with ACR increased from 35% to 73%) without an increase in adverse effects. However, at the maximal dose, there was no additional benefit (ACR in 65%), and two patients developed a prolonged QTc interval on their ECG. Thus an intermediate dose (B) was chosen as the safest and most effective to take forward into the next stage [32].

A voucher specimen of the plant harvested for making the phytomedicine was deposited in the herbarium of the Department for Traditional Medicine. Thin-layer chromatography of the plant extract (methanol) and of the decoction was used to identify already published constituents. This preliminary study was subsequently confirmed by HPLC and mass spectroscopy. Berberine and sanguinarine were detected in the methanol extract but sanguinarine was not detectable in the decoction. Lyophilized samples of the phytomedicine used were kept for reference, and future phytochemical fingerprinting.

Although according to WHO guidelines it would not have been necessary, the opportunity arose to conduct toxicological studies. These were conducted in parallel with the dose-escalating clinical trial. The LD50 of the freeze-dried decoction of the aerial parts was determined twice in two different laboratories which both showed that it was >3000mg/kg, as no rats or mice were adversely affected even by this high dose [33,34]. There is always a concern that some toxic ingredients are not absorbed in rodent species, but this was reassuring in the context of a long history of use of the leaf decoction in humans, with no reported toxicity.

## Stage 3: Randomized controlled trial (RCT)

As results from all previous stages were encouraging, the aim at this stage was to test the effectiveness of the phytomedicine in the field. In Mali, the objective was to develop a phytomedicine for home-based management of malaria (HMM), with the aims of symptomatic improvement and preventing severe malaria. The vision was that, if effective, the plant could be recommended to communities to be cultivated and prepared locally as a first-line treatment for presumed malaria. Therefore, the inclusion criteria for the RCT reflected this: all patients with presumed malaria (history of fever during the last 24 hours, without another obvious cause, during the rainy season) were included.

It is not ethical to give placebo or no treatment to the control group, because falciparum malaria is potentially fatal and can progress rapidly, particularly in non-immune patients. The most useful comparator is the nationally recommended first-line treatment. In most countries this is now an artemisinin combination therapy (ACT). In Mali it was artesunate-amodiaquine. Artemisinins are the most effective and rapid anti-malarial drugs ever discovered, so it is not realistic to aim for a herbal treatment to outperform an ACT. Rather the aim should be non-inferiority for the selected appropriate outcome measures, or at least reaching a certain pre-defined level of effectiveness.

The outcome measures are summarized in table 4 and the results in table 5. The primary outcome measure was “clinical recovery” at day 28, without the need for a second-line treatment. Over 28 days, second-line treatment was not required for 89% (95% CI 84.1–93.2) of patients on *A. mexicana*, versus 95% (95% CI 88.8–98.3) on artesunate-amodiaquine. An important secondary outcome measure was incidence of severe malaria, which is the most important outcome in public health terms. Large numbers of patients are needed in order to demonstrate non-inferiority, because severe malaria is a relatively uncommon event. However another approach is to see whether the incidence of severe malaria is kept below a pre-specified level in both groups [35]. In a previous study in a similar context, age-specific incidence (age <5 years) of severe malaria in untreated patients with presumed malaria was about 11%, and in patients treated at home with chloroquine (the standard treatment at that time) was about 5% per month [36]. The aim was, therefore, to keep the age-specific incidence of severe malaria (in patients aged <5 years) below 10%, and ideally ≤5%, in both groups. A sample of 300 patients was needed to answer this question (100 patients treated with ACT and 200 with *A. mexicana* decoction); an unequal randomization ratio was chosen in order to collect, with equal means, more information on the less known treatment. The observed age-specific incidence of severe malaria (in children aged 0-5 years) was 1.9% in both groups (*Argemone mexicana* and ACT) over the first 28 days of follow-up. The follow-up was extended to three months, and over this time the age-specific incidence of severe malaria was 2% per month in the herbal group and 1% per month in the ACT group. With 95% confidence, the age-specific incidence of severe malaria in both groups was <6% per month[37].

**Table 4:** Outcome measures used in a high-transmission area

Study	Primary Outcome	Secondary outcomes
Observational study: Dose-escalating clinical trial	% of patients with Adequate Clinical Response at d14 in each dosage group (= dose response)	% of patients with Adequate Clinical Response at d28
		% of patients with total parasite clearance at days 14 and 28
		% of patients experiencing adverse effects

Experimental study: Pragmatic Randomized Controlled Trial	‘clinical recovery’ at day 28 without need for re-treatment with the second-line anti-malarial	Axillary temperature <37.5’C at day 14
		Age-specific incidence of severe malaria days 0-28 (patients aged <5 years)
		incidence of new clinical episodes of malaria d15-28
		Mean haematocrit at day 28
		% of patients experiencing adverse events

**Table 5:** Oucomes of treatment of uncomplicated malaria by a village health worker with *Argemone mexicana* decoction (AM ) or Artemisinin Combination Therapy (ACT ) as first-line anti-malarial (% and 95%CI)[35].

	AM group	ACT group
No need for 2nd line treatment	89.3% (84.1 – 93.2)	95% (88.8 – 98.3)
T <37.5°C (**) at day 14	93.9 (89.3 - 96.7)	97.0 (91.6 – 99.4)
T <37.5°C at day 28	96.9 (93.5 – 98.9)	99.0 (94.6 – 100.0)
Severe malaria >5yo	0 (0 - 1.83)	0 (0 - 3.62)
Severe malaria (0-5 yo)	1.9% (0.2 – 6.7)	1.9% (0.05 – 10.3)
Severe malaria (all ages) - Coma / convulsions	0 (0 - 1.83)	0 (0 - 3.62)
Adverse effects	14.2% (9.7 - 19.9)*	18.8% (11.7 - 27.8)*
New episode (day 15-28) (parasite positive)	12.8% (8.4 – 18.3)	9.9% (4.9 – 17.5)

* Most common adverse effects : Cough and diarrhoea with AM, vomiting with ACT

** Temperature in degree Celsius

## Stage 4: Isolation and testing of active compounds

This is the last step of “reverse pharmacology”. A phytomedicine can be developed without isolating an active ingredient, but it is useful to do this for two reasons. First and foremost there needs to be a phytochemical marker for quality control and standardization of the herbal medicine, and also to permit agronomic selection of the best plants. Secondly it is possible that a new modern drug could be developed in parallel by the pharmaceutical industry. However it makes more sense to do this after the clinical safety and effectiveness have already been demonstrated, as chances may be higher that the isolated compound (or a derivative) will also be safe and effective. Much time and money is wasted in developing drugs which turn out to be unsafe or ineffective in humans [38].

Isolating pure active ingredients from a phytomedicine is not straightforward. Most phytomedicines contain several compounds with additive or synergistic activities, or even pro-drugs. *Argemone mexicana* contains at least three protoberberine alkaloids in similar amounts (around 0.5% in the plants from Mali) with similar anti-malarial activity: berberine, protopine and allocryptopine (IC50 in vitro = 0.32, 0.32 and 1.46 mcg/ml respectively) [39]. Whereas all are active *in vitro*, the absorption of berberine is poor in some animal models, although it can be improved by P-glycoprotein inhibitors [40]. It is not known whether *A. mexicana* contains any P-glycoprotein inhibitors, but if it does, their concentration would also be important. The pharmacokinetics of protopine and allocryptopine have not yet been studied in humans, so it is not known which of these is the best marker, or whether there is synergy between them (in which case maybe all should be used as markers). Unlike berberine, protopine and allocryptopine show a good selectivity for *Plasmodium* and their cytotoxicities are low [39]. Since preliminary *in vivo* tests using freeze dried AM decoction were unsuccessful both in mouse and in rat models (*Plasmodium berghei* and *Plasmodium chabaudi* respectively, unpublished results), the plan is now to study the *in vitro* antiplasmodial activity of plasma samples from healthy volunteers to identify plant substances or metabolites involved in such activity.

## Discussion

While developing new compounds from natural products could be an important source of new anti-malarials in the long term, it is also possible to develop standardized and validated phytomedicines more quickly and cheaply. The scheme used has already saved considerable time and money in developing a new herbal anti-malarial in Mali. Since the studied plant is a pan-tropical weed, results of such a research programme could be applied in many countries, provided there is local quality control of the plants.

It is of paramount importance to conduct such research in an ethical manner, and all the clinical trials were submitted and approved by an ethics committee. To be ethical, a non-inferiority trial needs to test a strategy that could be sustained after the end of the research. During the study there must be proper safeguards in place to ensure the safety of the patients, so a medical team was stationed in the village for the whole period to give immediate care when required. The result was to inform the villagers which of their traditional remedies has been clinically shown to be an effective anti-malarial, at what dose, what precautions are necessary in its preparation, and that they should rapidly seek modern medical treatment if they do not improve or if danger signs appear. It is likely that this knowledge is of more benefit in the long term, and so more ethical, than a short-term unsustainable intervention. This hypothesis will be tested in future research on the public health impact of such information.

With the benefit of hindsight, there are some improvements which could be made to the scheme. In the initial selection of the plant, the determining factor should be the observed treatment-effect correlation in the RTO rather than the *in vitro* activity, which can be misleading. In this case the *in vitro* activity was largely attributable to berberine, which is poorly absorbed, and is probably not (or not directly) responsible for the activity in humans.

In the dose-escalating study, it would have been better to start by consulting those familiar with the remedy about the minimum and maximum doses which patients can take, to ensure that the traditional healer was proposing his usual range of doses for the study. Based on this information, two or three different standard dosages of the phytomedicine could be defined in advance in the trial protocol. Of course, the same inclusion criteria must be used throughout the trial, so that the patients in each group are as similar as possible.

In other contexts the aim of treatment may be different. For example in Brazil, *Artemisia annua* infusion is being tested as a backup for situations in which the recommended first-line treatment is not available. These include stock-outs of standard drugs, and remote areas which are not reached by the healthcare infrastructure. Brazil is a low transmission area, and total parasite clearance is considered mandatory. In this context ACPR is the chosen outcome measure.

Other phytomedicines for malaria have already been developed and are government-approved in Burkina Faso (*Cochlospermum planchonii* root decoction), in Ghana (*Cryptolepis sanguinolenta* root infusion) [41], and in the Democratic Republic of Congo (*Artemisia annua* Anamed leaf infusion). Much of the development work has already been done on these: their safety has been demonstrated and they seem efficacious in preliminary clinical trials. However further work is needed to decide how they would fit into public health strategies for control or elimination of malaria. It is important to develop cheap and reliable tests for quality control and standardization of plant material. Larger scale clinical trials are needed, including children who are most at risk of severe malaria, if they are intended to be future users of a validated and officially recommended phytomedicine. This is not the case in Mali, where it has been proposed to test on a small scale a health policy including *Argemone mexicana* decoction for the home-based management of malaria in patients aged over five years in high transmission areas, thereby saving ACTs for children aged five years and under [35].

There is a range of other promising anti-malarial phytomedicines which could be developed much faster and more cheaply than new chemical compounds, because preliminary work has already provided some information on their safety and efficacy [42]. Such phytomedicines could be considered not only for treatment of malaria but also for prophylaxis and intermittent presumptive treatment. The proposed methodology could also be adapted to develop herbal prophylactics, starting from good ethnomedical observation and progressing though clinical studies (although the protocols would be different from those described here, which are designed to evaluate potential treatments).

Funding organizations should support the possibility of developing new types of medicines, including phytomedicines, rather than restricting funding only to conventional development of isolated active compounds. *Sustainable* public health improvement in remote areas is a key consideration in such a discussion. Innovative Public Private Partnerships could also be considered with companies already expert in the production of standardized phytomedicines.

## Competing interests

The authors declare that they have no competing interests.
